# Long horns protect *Hestina japonica* butterfly larvae from their natural enemies

**DOI:** 10.1038/s41598-022-06770-y

**Published:** 2022-02-18

**Authors:** Ikuo Kandori, Mamoru Hiramatsu, Minako Soda, Shinya Nakashima, Shun Funami, Tomoyuki Yokoi, Kazuko Tsuchihara, Daniel R. Papaj

**Affiliations:** 1 Faculty of Agriculture, Kindai University, Nara, 631-8505 Japan; 2 Faculty of Life and Environmental Sciences, University of Tsukuba, Tsukuba, Ibaraki 305-8572 Japan; 3 Department of Information Science, Tohoku Gakuin University, Sendai, Miyagi 981-3193 Japan; 4 Department of Ecology and Evolutionary Biology, University of Arizona, Tucson, AZ 85721 USA

**Keywords:** Behavioural ecology, Entomology

## Abstract

Animals sometimes have prominent projections on or near their heads serving diverse functions such as male combat, mate attraction, digging, capturing prey, sensing or defence against predators. Some butterfly larvae possess a pair of long frontal projections; however, the function of those projections is not well known. *Hestina japonica* butterfly larvae have a pair of long hard projections on their heads (i.e., horns). Here we hypothesized that they use these horns to protect themselves from natural enemies (i.e., predators and parasitoids). Field surveys revealed that the primary natural enemies of *H. japonica* larvae were *Polistes* wasps. Cage experiments revealed that larvae with horns intact and larvae with horns removed and fitted with horns of other individuals succeeded in defending themselves against attacks of *Polistes* wasps significantly more often than larvae with horns removed. We discuss that the horns counter the paper wasps’ hunting strategy of first biting the larvae’s ‘necks’ and note that horns evolved repeatedly only within the Nymphalidae in a phylogeny of the Lepidoptera. This is the first demonstration that arthropods use head projections for physical defence against predators.

## Introduction

Animals sometimes develop conspicuous projections on or close to their heads, such as horns, antlers, tusks, enlarged mandibles, barbels, and antennae. These projections can be largely divided into four groups according to their roles. First, some structures develop only in adult males and are used in intra-sexual contests for mates or in mate attraction. Such structures are numerous: horns, antlers and tusks in mammals^1–4^, horns and enlarged mandibles of some beetles^5–8^, and eye stalks of certain flies^9,10^. Second, structures such as protruding tusk-like teeth or horns are used as a tool for daily life such as to burrow or dig the soil in naked mole rats^11^ and the sand-living anthicid beetle *Mecynotarsus tenuipes*^12^ and to capture prey items in the larvae of the diving beetle *Hyphydrus japonicas*^13^. Third, some projections have many mechanical and chemical sensillae on their surface^14–17^ and are used as probes to locate resources such as food, host plants, and mates^18–20^. Such projections include barbels in fish and antennae in insects and other arthropods such as centipedes, millipedes, macrurans, hermit crabs, and pill bugs. Fourth, these structures may be used as anti-predator weaponry. Examples include horns of horned lizards and female bovids^21,22^. In another example, swallowtail butterfly larvae use eversible projections that temporarily protrude from just behind their heads for chemical defence against natural enemies^23,24^. However, head projections in insects and other arthropods that are specialized for physical defence against predators are not known.

**Figure 1:**
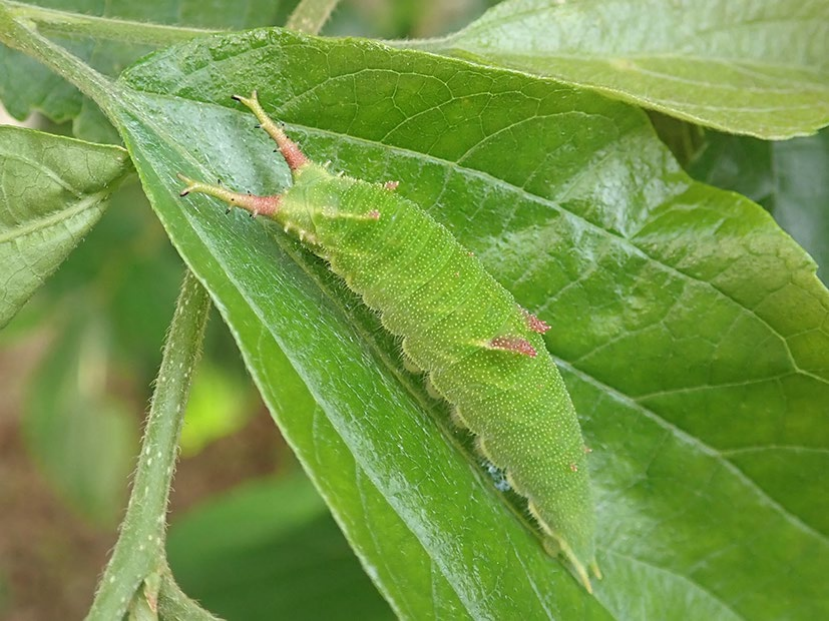
Last instar larva of *Hestina japonica*.

Some species of butterfly larvae have a pair of long projections that are fixed on or close to their heads (see Discussion for taxonomic distribution of larvae with horns). They look like antennae but are not; their actual antennae are small, short and three-segmented structures found close to their mouthparts^25,26^. The function of these projections is generally not well known. One exception is the use of projections by larvae of the pipevine swallowtail *Battus philenor* to locate and assess food^27^. Larvae of *Hestina japonica* (Nymphalidae) also have a pair of long projections. Unlike the elastic, fleshy, and mobile projections of *B. philenor* larvae that grow just behind their heads, the projections of *H. japonica* larvae are hard, attached directly to their heads, and incapable of bending, and will hereafter be referred as horns (Fig. 1). They have a pair of horns throughout their larval stage except the first instar.

In this study, we investigated the role of the horns of *H. japonica* larvae. We hypothesized that larvae use the horns as physical shields to protect themselves from natural enemies. In the first experiment, field surveys were conducted to reveal natural enemies of *H. japonica* larvae. In the second experiment, we examined whether the larvae effectively defend themselves against the attack of *Polistes* wasps, the primary natural enemies of *H. japonica* larvae, by using their horns as a physical shield.

## Results

### Experiment 1: field survey of natural enemies of *H. japonica* larvae

**Figure 2:**
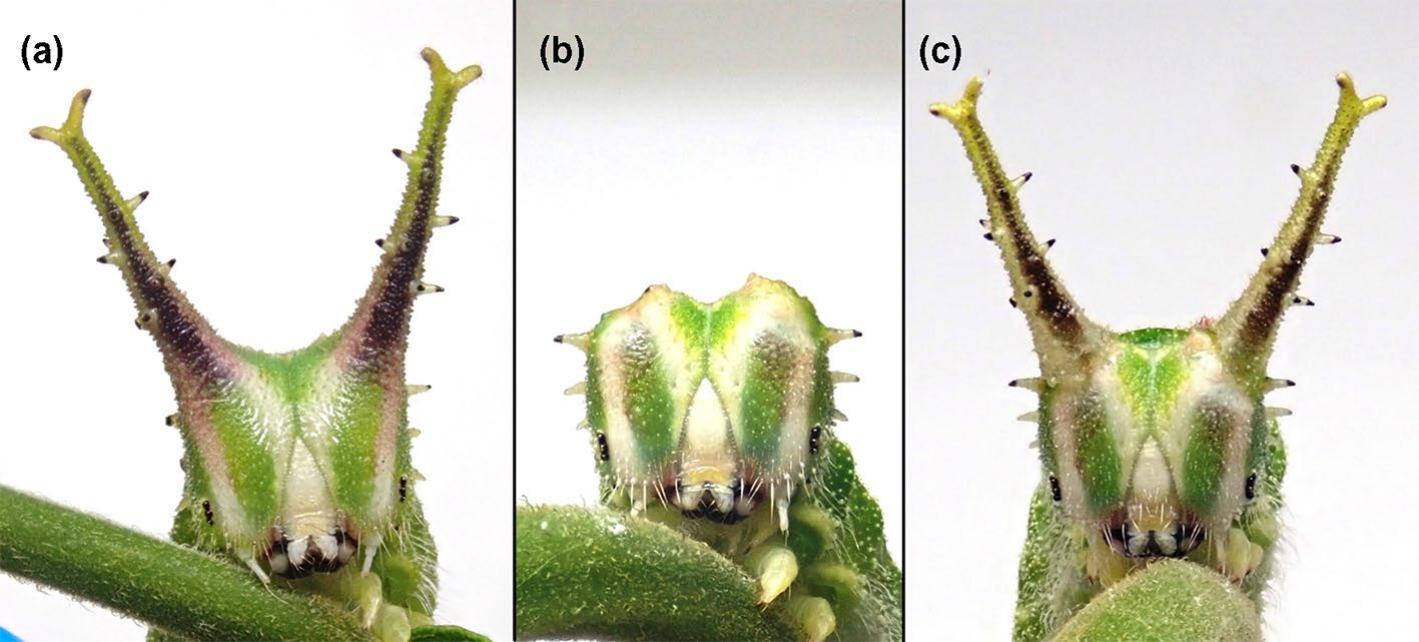
The frontal images of *Hestina japonica* larvae in three treatments used in Experiment 2. (**a**) normal larvae with horns intact, (**b**) larvae with horns removed, and (**c**) larvae with horns removed and then fitted with shed horns of different individuals.

Field observation by video camera recording was conducted in Mt. Ikoma and in the campus of Kindai University. During about 500 h of filming towards approximately 140 individual larvae, we observed a total 121 attacks on *H. japonica* larvae by natural enemies (Table 1). Almost all larvae stayed motionless on the upper side of the leaves of host trees when they were attacked (see Fig. 1). *Polistes* wasps, consisting mainly of *P. jokahamae* and *P. japonicus*, were the most common attackers, accounting for 86.8% of attacks. The second most common natural enemy were birds, which accounted for 8.3% of attacks. Two-thirds of the larvae survived the attack of *Polistes* wasps, whereas none of them survived the attack of birds (Table 1, Supplementary Video S1 and S2) (Fig. 2).

**Table 1:** Natural enemies attacking *Hestina japonica* larvae and their survival just after being attacked in the field.

Natural enemy	Attack		Survival	% Survival^c^
	*N*	(%)	*N*	
Insecta				
Hymenoptera				
*Polistes jokahamae* (Vespidae)	56	(46.3)	30	53.6
*Polistes japonicus* (Vespidae)	24	(19.8)	20	83.3
*Polistes rothneyi* (Vespidae)	14	(11.6)	9	64.3
*Polistes mandarinus* (Vespidae)	2	(1.7)	2	100.0
*Polistes snelleni* (Vespidae)	1	(0.8)	1	100.0
*Polistes chinensis* (Vespidae)	1	(0.8)	1	100.0
*Polistes* spp. (Vespidae)^a^	7	(5.8)	7	100.0
*Vespula flaviceps* (Vespidae)	3	(2.5)	3	100.0
*Formica japonica* (Formicidae)	1	(0.8)	1	100.0
Diptera				
Parasitic fly gen. sp.^b^	1	(0.8)	1	100.0
Arachnida				
Araneae				
*Myrmarachne* sp. (Salticidae)	1	(0.8)	1	100.0
Aves				
Passeriformes				
*Parus minor* (Paridae)	8	(6.6)	0	0.0
*Emberiza cioides* (Emberizidae)	2	(1.7)	0	0.0
Subtotals				
Hymenoptera				
Polistes	105	(86.8)	70	66.7
Other hymenoptera	4	(3.3)	4	100.0
Diptera	1	(0.8)	-	
Araneae	1	(0.8)	1	100.0
Passeriformes	10	(8.3)	0	0.0
Total	121	(100.0)	75	62.0

^a^Includes all *Polistes* wasps whose species could not be identified due to focus or subject distance from the video camera.

^b^The fly laid some eggs on the larva. The long-term survival of the parasitized larva was not tracked.

^c^Percentage of attacks that larvae survived.

### Experiment 2: survival of *H. japonica* larvae after attack of *P. jokahamae* wasps

**Figure 3:**
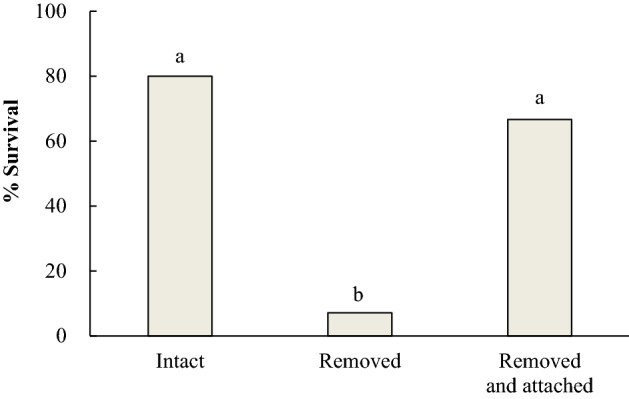
Survival of *Hestina japonica* larvae in three treatments upon being attacked by *Polistes jokahamae* wasps. The treatments were unmanipulated larvae with horns intact (treatment 1, left bar), larvae with horns removed (treatment 2, middle bar) and larvae with horns removed and horns of different individuals attached in their place (treatment 3, right bar). Different letters above the bars indicate significant differences between larval treatments (post hoc Bonferroni: *P* < 0.05; see Results for details).

Larvae were experimentally manipulated and randomly assigned to one of three treatments: the larvae with horns intact, the larvae with horns removed, and the larvae with horns removed upon which horns of different individuals were attached. They were subsequently placed inside the outdoor cages where *P. jokahamae* wasps were allowed to attack them. When the wasps attacked *H. japonica* larvae, most of the larvae simply turned their heads toward the wasps. They occasionally counterattacked by biting and striking the wasps with their horns. When larvae failed to defend themselves from wasp attack, the wasps always bit the larvae on the ‘neck’ (just behind the head capsule). Among the three treatments, larvae with horns intact (normal larvae) and larvae with their own horns removed and then fitted with horns from different individuals often succeeded in defending themselves from wasp attack (Fig. 3, Supplementary Video S3). In contrast, larvae with horns removed often failed to defend themselves, i.e., they were bitten on the neck and killed by the wasps (Fig. 3, Supplementary Video S4). Generalized Linear Mixed Models (GLMMs) testing for effects on survival of *H. japonica* larvae soon after being attacked by *P. jokahamae* wasps indicated that there was a significant effect of larval treatment and a nonsignificant effect of larvae size, year, and wasp nest (Table 2). Post hoc comparisons by t-test for pairwise contrasts and the sequential Bonferroni adjustment indicated that the two treatments of larvae that have a pair of horns either of their own (treatment 1, *N* = 15) or of other individuals (treatment 3, *N* = 14) did not differ in frequency of survival, and that larvae of each of those treatments survived at significantly higher frequencies than ones without horns (treatment 2, *N* = 15) (treatment 1 vs 2: t = 2.901, *P* = 0.018; treatment 2 vs 3: t = 2.629, *P* = 0.025; treatment 1 vs 3: t = 0.499, *P* = 0.621; Fig. 3). These results suggest that horns of *H. japonica* larvae are effective in defending themselves against wasp attacks. The fact that larvae with re-attached horns did not suffer decreases in survival relative to unmanipulated larvae suggests that any harm done to larvae by artificially removing their horns did not account for the effect of removal on survival to attacks.

**Table 2:** Summary of GLMM to test for effects on survival of *Hestina japonica* larvae immediately after being attacked by *Polistes jokahamae* wasps.

Fixed effects:	F	*P*
Larval treatment	F_2,38_ = 4.477	**0.018**
Larval size	F_1,38_ = 1.058	0.310

‘Larval Treatment’ refers to the effect of three treatments of larvae, i.e., normal larvae with horns intact, larvae with horns removed, and larvae with horns removed and then fitted with shed horns of different individuals. ‘Larval Size’ refers to the effect of body length of larvae. Significant *P*-values are in bold.

## Discussion

Butterfly larvae sometimes develop a pair of long projections on or close to their heads. This study demonstrated that *H. japonica* larvae use a pair of long and hard head projections, i.e., horns, to protect themselves physically from *P. jokahamae* paper wasps, their main natural enemies. While it has been known that some vertebrates use horns as a physical shield to defend against natural enemies^21,22^, this study provides the first example of such defence for an invertebrate.

Morphological defence of lepidopteran larvae against natural enemies includes hairs and spines covering the entire body, hard epidermis, mimicry of tree branches, moss and bird droppings, and aposematic or cryptic coloration^28,29^. Our study added a new method of morphological defence of lepidopteran larvae, i.e., using horns as a physical shield.

According to observations in a preliminary experiment, *Polistes* wasps often bite on the lepidopteran larvae's ‘neck’ (just behind the head capsule) during the attack, like a lion bites on the prey's neck (Supplementary Video S5). We could not find any previous literature that shows paper wasps or hornets adopting similar hunting strategies. However, field video filming in Experiment 1 confirmed this hunting strategy of *Polistes* wasps. In a trial in which the paper wasps succeeded in attacking (that is, the larva failed to defend), the body parts of the larva that the paper wasps first bite (that is, that the hemolymph of larvae first came out) were counted. As a result, the three main species of *Polistes* wasps bit the larval neck almost without exception (21 out of 22 trials for *P. jokahamae*, 3 out of 3 trials for *P. japonicus* and 3 out of 3 trials for *P. rothneyi*, when we extracted only trials of filming in which the larval body part bitten was clearly visible).

This hunting strategy may allow the wasps to cut the larvae's head and incapacitate the larvae without risk of a counterattack. Observations in Experiment 2 indicated that larvae in all three treatments simply turned their heads toward the attacking wasps. This behavioural response seems to make it difficult for the wasps to bite on the neck of larvae that have horns. Therefore the horns of *H. japonica* larvae served as defensive shields to protect their neck.

Are the larval horns effective against natural enemies other than paper wasps? Results of Experiment 1 suggest that they are not effective against birds at all. Due to the large difference in body size, the larvae seemed unable to resist attack by the birds and were taken as prey. Spiders, predatory bugs, ants, mantids, parasitoid wasps and parasitoid flies may also be natural enemies of these larvae^28^. However, they were rarely seen in the video collected in Experiment 1, or even if they appeared, they did not come into contact with the larvae. Thus, we do not know whether the larval horns are effective against these natural enemies.

It is known that the larger the size of the prey, the more effective the prey is in defending itself against natural enemies^30^. However, in Experiment 2, larval body size did not significantly affect larval survival to attacks. The reason may be that we used larvae of a relatively limited body size range (specifically sizes typical for larvae in the middle of the last instar). Perhaps if we used larvae of more instars, resulting in a greater range of sizes, we might have detected an effect of body size on survival.

In closing, the long frontal projections of butterfly larvae that are fixed on or near their heads may be roughly divided into two types: soft ones that grow just behind the head capsule and hard ones that grow directly on the head capsule (i.e., horns). As far as the latter type is concerned, it appears to be found in at least nine of the 12 subfamilies of Nymphalidae, i.e., all genera belonging Pseudergolinae, Apaturinae (including *H. japonica*), Cyrestinae and Calinaginae, most genera belonging Biblidinae and Charaxinae, and some genera belonging Heliconiinae, Nymphalinae and Satyrinae (Supplementary Table S1 and S2)^31–77^. To the best of our knowledge, larval horns seem to have evolved repeatedly within the Nymphalidae and nowhere else in the Lepidopteran phylogeny. By studying additional butterfly species, we are currently testing the hypothesis that the former type of projection may be widely used for host plant search, as shown for *B. philenor*^27^, and the latter for defence against natural enemies, as shown in this study for *H. japonica*.

## Methods

### Study species

*Hestina japonica* (C.& R. Felder) is distributed in East Asia including Japan. Larval host plants include Japanese hackberry *Celtis sinensis* and several species within the same genus *Celtis* (Family Ulmaceae). In Japan, *H. japonica* adults are common between May and August. Larvae have a pair of horns protruding from the head capsule from the second to the last (sixth) instar (Fig. 1). In March 2015, 2017, and 2018, overwintering penultimate-instar larvae of this species were collected from the fallen leaves near the roots of *C. sinensis* trees in Mt. Ikoma (Ikoma, Nara prefecture), in the campus of Kindai University (Nara, Nara prefecture), and in Kizugawa riverbed (Kyotanabe, Kyoto prefecture). From June to September of the same years, eggs and larvae of various instars were also collected from *C. sinensis* leaves in the latter two places mentioned above. Each larva was placed in a 430 ml transparent plastic cup, fed with fresh *C. sinensis* leaves, and maintained at 25 °C and a light/dark (L:D) period of 13 h:11 h in an incubation room, and used for Experiments 1 and 2.

Following the results of Experiment 1, the paper wasp, *Polistes jokahamae* Radoszkowski was used in Experiment 2. This species is the primary natural enemy of *H. japonica* larvae. A particularly large and aggressive species of *Polistes* wasp, it is distributed throughout East Asia including Japan^78^. Like many other *Polistes* species, this species has an annual colony cycle, with each nest founded by one mated foundress in mid-spring. The adults not only feed on floral nectars, honeydew of aphids and ripe fruits, but also capture and process caterpillars and other invertebrate prey to feed to larvae back at the nest^78^. The method of collecting and maintaining the wasps will be explained in the methods section for Experiment 2.

### Experiment 1: field survey of natural enemies of *H. japonica* larvae

To determine the primary natural enemies of *H. japonica* larvae, field observation by video camera recording was conducted from 8:00–9:00 to 17:00–18:00 for 55 days from April to September 2017 in Mt. Ikoma and in the campus of Kindai University. We selected *C. sinensis* trees in which we had previously found larvae or eggs of *H. japonica* (one site in Mt. Ikoma and three sites in the campus of Kindai University). The day before observation, we released one or several butterfly larvae at the stage of mainly last instar and some penultimate instar on branches of host trees at a height of 1.0–1.5 m that were covered with fine nylon netting (ca. 30 cm in diameter). In the morning of the observation day, the netting was removed. The larvae usually stayed motionless on the upper side of the leaf during this procedure. We then set a video camera (JVC GZ-R400) on a tripod 2–3 m away from the larvae. The camera angle was adjusted so that one to several larvae could be monitored on the screen and video recording was started (resolution: 1920 × 1080 pixels; frame rate: 30 fps). After each day of video recording, insects and other animals in the video were identified to species, wherever possible. When potential natural enemies (either predators or parasitoids) encountered the larvae and elicited a reaction, such as the larvae turning their heads to the contacted side or shaking their heads from side to side, they were considered to have attacked the larvae. The survival of the larvae soon after each attack was also recorded (whether larvae succeeded in defending and survived, or failed to defend and were killed). We could not find nocturnal natural enemies attacking the larvae during a few days of preliminary observation at night (I. Kandori, personal observation).

### Experiment 2: survival of *H. japonica* larvae after attack of *P. jokahamae* wasps

To find out whether the larvae use the pair of horns for defence, we conducted larval predation experiments, using *Polistes jokahamae* as the predator. *P. jokahamae* was found to be the most abundant natural enemy in the field survey of Experiment 1.

### Preparation of wasps

From May to June in 2015 and 2018, nests of *P. jokahamae* wasps were collected around the campus of Kindai University, Nara. We collected early-stage small nests, generally containing 10–20 cells with a single foundress and numerous pupae, larvae and eggs. After temporarily isolating the foundress, the nest was attached with glue to a piece of cardboard (5.0 × 5.0 cm). The cardboard was attached with double-sided tape to the ceiling of a plastic container (W22.5 × D14.5 × H14.0 cm) whose opening was at the side of the container. The container was placed in the corner of an outdoor cage (1.8 × 1.8 × 1.8 m). The container was kept at a height of about 50 cm by placing it atop a stack of concrete blocks. The foundress was then returned to the nest. Next, insect jelly (Marukan, Flat 55) and old wooden stakes (8 cm in diameter, 100 cm long) were placed in the outdoor cage as food for adults and nesting material, respectively. In addition, several potted plants of *C. sinensis* (about 100 cm high) were placed into the outdoor cage. As food for larval provisioning by the wasps, we used 1 to 3 silk moth larvae with a body length of about 30 mm each day. Just before feeding larvae to the wasps, we disabled them by crushing the head capsules with tweezers. Then we placed them on the leaves of potted *C. sinensis*. In this way we fed the wasps without allowing them the experience of hunting live larvae and the wasps were conditioned to forage on *C. sinensis* plants. Multiple nests were maintained simultaneously during the experimental season, with one nest per one outdoor cage. After more than one month of bringing the nest into the cage, when the nest contained a foundress and more than several workers, we began the assay of wasp attack by using only workers (see below). For individual identification, each worker was marked with a different colored paint marker on the back of the thorax before or immediately after attacking the *H. japonica* larvae in the assay. When marking workers, we temporarily trapped the wasps in plastic cups and anesthetized them with carbon dioxide.

### Preparation of three treatments of *H. japonica* larvae

To verify the hypothesis that *H. japonica* larvae use the horns as shields to protect themselves from their main predator, we prepared three different treatments of last-instar larvae: (1) larvae with horns intact (normal larvae); (2) larvae with horns removed and; (3) larvae with horns removed upon which horns of different individuals were attached (Fig. 2). Preliminary trials revealed that, when we cut the horns of the larva directly with a dissection scissors, they lost a large amount of hemolymph and became weak. By using the following method, we successfully removed horns with minimal damage to the larva. The larvae of the penultimate instar were first temporarily paralyzed by chilling on an ice pack. Next, the tips of forceps were heated with a gas burner until they turned red. The heated forceps were used to pinch and burn the central part of the horns. After that, the larvae were returned to normal rearing. Usually, they successfully molted to the last instar and lost their horns without any obvious loss of hemolymph (see Supplementary Figure S1). We set up the third treatment of larvae, to control for the potential trauma caused by the artificial removal of their horns. This treatment of larvae was produced by removing the horns as described above, and by then attaching a pair of horns with instant adhesive. The new horns were recovered from conspecifics that shed them when they pupated. Mid-last instar larvae (24–33 mm in body length) were used in the experiment.

### Assay of wasp attacks

The assay of attacks on treated larvae was carried out in the outdoor cage where paper wasps were maintained. The *H. japonica* larvae in each treatment were measured for their body length (from head capsule excluding horns to the end of abdomen) and marked individually with a colored paint markers on their dorsal surface. The day before experiment, larvae were placed on the leaf of potted *C. sinensis* and covered with a fine meshed bag. On the day of experiment, after we confirmed that larvae stayed still on the upper surface of the leaf, we uncovered the bag and larvae were exposed to foraging worker wasps. When the wasp attacked a larva, we investigated whether the larva succeeded in defending itself or not, that is, whether the wasp gave up and flew away (= survival), or the larva failed to defend itself and was killed by the wasp (= death). The defence success (% survival) was compared among the three treatments of *H. japonica* larvae. Only the first attack was recorded for each wasp and for each larva, indicating that the wasps and the larvae were inexperienced with hunting and being attacked, respectively. Experiments were conducted from July to September in 2015 and 2018. In each year, paper wasps from three nests were used in the experiment.

### Statistical analysis

We used generalized linear mixed models (GLMMs) with type III sums of squares to test for effects on survival of *H. japonica* larvae soon after being attacked by *P. jokahamae* wasps with a binomial error distribution and a logit link. Survival or death (1/0) of *H. japonica* larvae was used as the binary response variable. Larval treatment and larval body length were treated as fixed effects. Year (two years of 2015 and 2018) and wasp nest (five nests for two years) were treated as random effects. When the larval treatment had a significant effect, post hoc multiple comparisons were performed among estimated marginal means of the three treatments by t-tests for pairwise contrasts and the sequential Bonferroni adjustment. IBM SPSS statistics 25 was used for all statistical analyses^79^.

## Supplementary Information

Supplementary Tables.

Supplementary Figure S1.

Supplementary Legends.

Supplementary Table S1.

Supplementary Table S2.

Supplementary Video S1.

Supplementary Video S2.

Supplementary Video S3.

Supplementary Video S4.

Supplementary Video S5.
