# Supplementary material for: Long horns protect Hestina japonica butterfly larvae from their natural enemies
Source: Sci Rep. 2022 Feb 18;12:2835. doi: 10.1038/s41598-022-06770-y (PMC8857287; doi:10.1038/s41598-022-06770-y)
Supplement: Supplementary file 2 — Supplementary Figure S1. [file 41598_2022_6770_MOESM2_ESM.docx]

**Supplementary Information - Fig. S1**


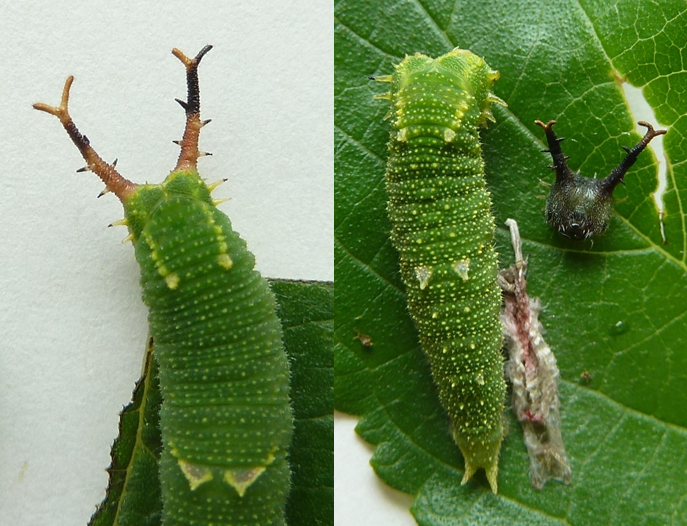


Fig. S1 The process of artificially removing horns of *Hestina japonica* larvae. The heated forceps were used to pinch and burn the central part of the horns of a penultimate-instar larva (left). After that, the larva molted to the last instar and lost their horns without any obvious loss of hemolymph (right).
