# Supplementary material for: Long horns protect Hestina japonica butterfly larvae from their natural enemies
Source: Sci Rep. 2022 Feb 18;12:2835. doi: 10.1038/s41598-022-06770-y (PMC8857287; doi:10.1038/s41598-022-06770-y)
Supplement: Supplementary file 3 — Supplementary Legends. [file 41598_2022_6770_MOESM3_ESM.docx]

**Supplementary Information – video legends**

Video S1 A paper wasp, *Polistes japonicus,* attacking the last-instar of *Hestina japonica* larva in the field survey of natural enemies in Experiment 1. The larva succeeded in defending and survived.

Video S2 A bird, *Parus minor*, attacking the last-instar of *Hestina japonica* larva in the field survey of natural enemies in Experiment 1. The larva failed to defend and was taken away.

Video S3 *Polistes jokahamae* wasp attacking the last-instar of *Hestina japonica* larva with horns intact in Experiment 2. The larva succeeded in defending and survived.

Video S4 *Polistes jokahamae* wasp attacking the last-instar of *Hestina japonica* larva whose horns were removed in Experiment 2. The larva failed to defend and was killed.

Video S5 *Polistes japonicus* wasp attacking the moth larva, *Xanthodes transversa*, in a preliminary experiment. The wasp mistakenly tried to bite at the end of larval body first, but soon re-bit at the ‘neck’ of the larva.
