# Supplementary material for: Long horns protect Hestina japonica butterfly larvae from their natural enemies
Source: Sci Rep. 2022 Feb 18;12:2835. doi: 10.1038/s41598-022-06770-y (PMC8857287; doi:10.1038/s41598-022-06770-y)
Supplement: Supplementary file 4 — Supplementary Table S1. [file 41598_2022_6770_MOESM4_ESM.docx]

**Supplementary Table S1.** Number of genera in which the larvae are horned or hornless on the head capsule within each Nymphalidae subfamily. See Supplementary Table S2 for more detail.

^a^ Horned larvae include ‘long-horned’ and ‘short-horned’ larvae in Table S2.
